# Prediction of Cardiopulmonary Resuscitation Outcomes for Arrest in Surgical Settings

**DOI:** 10.1001/jamanetworkopen.2025.39767

**Published:** 2025-10-28

**Authors:** Lucy Chen, Samuel Justice, Matthew B. Allen

**Affiliations:** 1 Department of Anesthesiology, Mass General Brigham, Harvard Medical School, Boston, Massachusetts; 2 Mass General Brigham AI, Boston, Massachusetts

## Abstract

**Importance:**

Perioperative cardiac arrest and cardiopulmonary resuscitation (CPR) are associated with significant morbidity and mortality. Despite a growing focus on goal-concordant surgical care and longstanding emphasis on preoperative code status discussions, clinicians lack tools to individualize risk estimates and inform shared decision-making (SDM) regarding perioperative CPR.

**Objective:**

To generate and internally validate predictive models for 30-day mortality and nonhome discharge using routinely available preoperative data.

**Design, Setting, and Participants:**

A prospective, multicenter, prognostic study of patients within the American College of Surgeons (ACS)-National Surgical Quality Improvement Program (NSQIP), including nearly 700 participating hospitals in the US, from January 1, 2012, through December 31, 2023. Follow-up duration was 30 days. Seven machine learning models were developed using 10-fold cross validation. Participants were patients aged 18 years or older undergoing noncardiac surgery who underwent CPR on the day of surgery.

**Exposures:**

Thirty-three preoperative sociodemographic, clinical, laboratory, and procedural variables were evaluated for their association with 30-day mortality and nonhome discharge.

**Main Outcomes and Measures:**

The primary outcome was 30-day mortality following CPR. The secondary outcome was nonhome discharge among survivors admitted from home. Performance was evaluated using area under the receiver operating characteristic curve (AUROC), accuracy, sensitivity, specificity, positive predictive value, negative predictive value, and calibration (Brier score and calibration curves). Clinical utility was evaluated using Shapley additive values (SHAP) decision curve analysis (DCA).

**Results:**

Among 6405 patients (median [IQR] age 69 [60-78], 3572 [55.8%] men, 860 [13.4%] Black, 4343 [67.8%] White, and 261 [4.1%] another racial category, including American Indian or Alaska Native, Asian, and Native Hawaiian or Other Pacific Islander), 3710 (57.9%) died within 30 days. The extreme gradient boosting model (CPR Outcome Prediction for Arrest in Surgical Settings [COMPASS]) achieved the best performance (AUROC for mortality = 0.80; 95 % CI, 0.78-0.82; accuracy = 0.73; 95% CI, 0.71-0.75; sensitivity = 0.77; 95% CI, 0.74-0.79; specificity = 0.68; 95% CI, 0.65-0.71; Brier score = 0.18). Among 2478 survivors admitted from home, 822 (33.2%) were discharged to a facility. For nonhome discharge, extreme gradient boosting demonstrated an AUROC of 0.78 (95 % CI, 0.74-0.82), accuracy of 0.76; and Brier score of 0.17. SHAP analysis identified American Society of Anesthesiologists status, case urgency, and frailty as key predictors. DCA indicated greater net benefit of extreme gradient boosting over default strategies (ie, treat all or treat none) across wide threshold ranges.

**Conclusions and Relevance:**

In this prospective prognostic study of outcomes following perioperative CPR, extreme gradient boosting generated individualized predictions of outcomes following perioperative CPR that may inform prevention strategies and goal-concordant surgical care.

## Key Points

**Question:**

Can a machine learning model using preoperative data predict outcomes following cardiopulmonary resuscitation (CPR) for perioperative cardiac arrest?

**Findings:**

In this prognostic study of 6405 perioperative cardiac arrests, models successfully predicted mortality and nonhome discharge using preoperative data. Extreme gradient boosting demonstrated the best performance, achieving areas under the receiver operating characteristic curve of 0.80 and 0.78 for mortality and nonhome discharge, respectively, as well as favorable predictive accuracy, calibration, and decision curve analysis.

**Meaning:**

These findings suggest a machine-learning model using preoperative data provided patient-specific predictions of outcomes following perioperative CPR and may inform preventive strategies and shared decision-making.

## Teaser

This prognostic study generates and internally validates predictive models for 30-day mortality and nonhome discharge using routinely available preoperative data.

## Introduction

Perioperative cardiac arrest is a serious complication of surgery and anesthesia associated with significant morbidity and mortality.^1,2,3,4,5,6^ Although rare (affecting approximately 5 in 10 000 patients),^7^ cardiac arrest in the setting of surgery is more common in high-risk groups, occurring in approximately 1 in 1200 older adults with frailty.^8^ For decades, the American Society of Anesthesiologists (ASA) and American College of Surgeons (ACS) have recommended preoperative discussion regarding the appropriateness of perioperative cardiopulmonary resuscitation (CPR) in patients with directives limiting treatment.^9,10,11,12^ More recently, the ACS and the American Geriatrics Society have extended this guidance to include all older patients regardless of prior code status.^13,14^ These initiatives reflect a growing emphasis on shared decision-making (SDM) regarding perioperative CPR—a process that requires evidence-based prediction of likely outcomes.^15,16^

Observational studies have estimated that perioperative CPR survival ranges from 32.0% to 55.7% at 24 hours^3^ (compared with roughly 25% following in-hospital arrest in nonsurgical settings).^17^ These studies have also identified conditions associated with worse outcomes following CPR for perioperative cardiac arrest (eg, ASA physical status, sepsis or septic shock, and emergency surgery),^4,18^ but the predictive accuracy of these features is poorly defined.^19^ Because perioperative code status discussions aim to tailor care to the individual,^9,10,11,12,20^ population-level estimates offer limited guidance; goal-concordant care instead requires tools that predict individual likelihood of benefit from potential interventions.^15^

Advances in machine learning (ML) have enabled the development of increasingly sophisticated predictive models for surgical outcomes and enhanced existing risk stratification tools.^21,22,23,24,25,26^ The only prior study we know of applying these methods to perioperative CPR was limited by small sample size and incomplete reporting of model performance.^27^ Applying ML methods to a large, national dataset may clarify the prognostic value of preoperative data and yield clinically useful predictions of outcomes following perioperative cardiac arrest. Such models could guide prevention strategies and inform preoperative SDM regarding appropriateness of CPR. The objective of this study was to generate and internally validate predictive models for 30-day mortality and nonhome discharge following CPR for perioperative cardiac arrest.

## Methods

### Study Design and Data

This prognostic study developed and internally validated predictive models for 30-day mortality and nonhome discharge following perioperative CPR using regression and ML methods. We used deidentified data from the 2012 to 2023 ACS National Surgical Quality Improvement Program (ACS NSQIP) including nearly 700 US hospitals, which was collected via trained medical record review. NSQIP methods, including rigorous quality control and database characteristics, have been described previously.^28^

We split our data into 70% training and 30% test datasets to internally validate the models. This study adhered to the Transparent Reporting of a Multivariable Prediction Model for Individual Prognosis or Diagnosis—Artificial Intelligence (TRIPOD+AI) reporting guideline and was deemed exempt from review and the need for informed consent due to the use of deidentified data by the Mass General Brigham institutional review board.^29^

### Participants

Our sample included patients aged 18 years or older undergoing noncardiac surgery who experienced cardiac arrest and underwent CPR on postoperative day (POD) 0 (ie, intraoperatively or postoperatively on the day of surgery). ACS-NSQIP defines cardiac arrest as “the absence of cardiac rhythm or presence of chaotic cardiac rhythm…which results in a cardiac arrest requiring the initiation of CPR.”^28^ Patients were excluded only if data were missing to establish outcome (eFigure in Supplement 1).

### Sample Size

We used a published sample size calculator for predictive models to determine the minimum sample size required a priori.^30^ Based on an event rate of about 60% for the primary outcome of mortality within 30 days,^19^ 33 candidate model features, and a target area under the receiver operating characteristic curve (AUROC) of at least 0.7 (deemed to represent acceptable discrimination),^31^ the minimum sample size needed was 2368.

### Outcome

The primary outcome was defined as mortality within 30 days of perioperative cardiac arrest and resuscitation. The secondary outcome was discharge to a destination other than home—such as a rehabilitation facility, skilled nursing facility, or long-term acute care hospital—among patients who were admitted from home.

### Candidate Predictors

Candidate predictors for 30-day mortality included 33 patient characteristics collected preoperatively. Sociodemographic characteristics included age, sex, and self-reported race and ethnicity (American Indian or Alaska Native, Asian, Black, Native Hawaiian or Pacific Islander, White, other, or unknown or not reported) to assess for possible predictive contribution. Clinical characteristics included admission from home vs other location, ASA physical status, frailty as measured by the revised Risk Analysis Index (RAI),^32,33^ functional status, body mass index, smoking status, ventilator dependence, weight loss, diabetes, dyspnea, chronic obstructive pulmonary disease, sepsis or septic shock, cancer, ascites, hypertension on medication, congestive heart failure, recent transfusion, bleeding disorder, dialysis, and acute kidney failure. Laboratory values included preoperative values for hematocrit, platelet count, white blood cell count (WBC), serum sodium, serum blood urea nitrogen (BUN), and serum creatinine. Surgical characteristics included procedure urgency (emergent, urgent, and elective), operative stress score (OSS) (low, 1-2; medium, 3; and high, 4-5),^34^ and year to adjust for secular trends. Characteristics of the subgroup analyzed for nonhome discharge are summarized in eTable 1 in Supplement 1. Intraoperative variables are not included in the NSQIP and were not analyzed.

### Data Preparation and Quality Checks

Our prespecified analysis plan included an assessment of missingness for potential variables to be included. All candidate predictors mentioned previously had a missingness rate below 10%, except for weight loss, dyspnea, and frailty (23.5%, 23.5%, and 25.4%, respectively) due to unavailability of these variables from 2021 to 2023. Missingness was addressed by imputation using bagging,^35^ and we additionally conducted a sensitivity analysis excluding 2021 to 2023 data (eTable 2 in Supplement 1).

### Model Development

Candidate predictors were defined before analysis. Analysis was performed March 1, 2024, through April 1, 2025, using R version 4.4.3 (R Project for Statistical Computing). We developed 7 models: random forest, extreme gradient boosting, neural network, naive Bayes, support vector machine, LASSO regression, and logistic regression. All models used 10-fold cross validation.

### Statistical Analysis

First, we compared our model, CPR Outcome Prediction for Arrest in Surgical Settings (COMPASS), output for the primary outcome of 30-day mortality with the ACS-NSQIP predicted mortality. We then assessed model discrimination by calculating AUROC with 95% CIs. Next, we calculated accuracy, sensitivity, specificity, positive predictive value, and negative predictive value with 95% CIs. Finally, we assessed model calibration by generating calibration plots and calculating Brier scores, which measure the agreement between predicted probabilities and observed outcomes. The same process was repeated for the secondary outcome, nonhome discharge. The optimal model for each outcome was then selected based on overall balance of performance metrics.

Clinical utility of specific features was examined using Shapley additive explanation (SHAP) values, which measure the change in prediction when a covariate is included or omitted for specific predictive outputs.^36,37^ We additionally performed decision curve analysis (DCA) to characterize clinical utility of the model as a whole compared with treat all and treat none default strategies.^38,39^ DCA evaluates the clinical usefulness of a predictive model by characterizing the value of the information it provides across a range of preferences regarding the relative burdens of undertreatment vs overtreatment. Such preferences are represented by the threshold probability (plotted on the x-axis), defined here as the predicted probability of a negative outcome beyond which a patient or clinician may reconsider the decision to proceed with CPR. The value of the information provided by the model at each threshold is quantified by net benefit (plotted on the y-axis), calculated as the proportion of true positives minus the proportion of false positives, weighted by the odds of the selected threshold probability.

## Results

Of the 10 724 265 noncardiac surgical cases in the ACS-NSQIP between 2012 and 2023, 6409 involved cardiac arrest on the day of surgery (incidence 0.06% or approximately 6 per 10 000 cases). After excluding 4 patients due to missing NSQIP predicted mortality or outcome, 6405 remained in the cohort (eFigure in the Supplement 1). The median (IQR) age was 69 (60-78); 3572 patients (55.8%) were men. In terms of race, 860 (13.4%) were Black, 4343 (67.8%) were White, and 261 (4.1%) identified with another racial category, including American Indian or Alaska Native, Asian, and Native Hawaiian or Other Pacific Islander. A total of 2269 (35.5%) and 1014 (15.9%) patients had ASA statuses of 4 and 5, respectively. Additional demographic and clinical characteristics are summarized in Table 1. Characteristics of the subgroup analyzed for nonhome discharge are summarized in eTable 1 in Supplement 1.

**Table 1.** Patient Characteristics and Candidate Predictors for 30-Day Mortality Following Perioperative Cardiac Arrest

Variable	Patients, No. (%)		SMD	Missing (%)
	Alive (n = 2695)	Dead (n = 3710)		
Age, y				
18-49	386 (14.3)	289 (7.8)	0.358	0
50-64	846 (31.4)	856 (23.1)		
65-74	740 (27.5)	1069 (28.8)		
75-84	514 (19.1)	978 (26.4)		
85	209 (7.8)	520 (14.0)		
Sex				
Female	1205 (44.7)	1628 (43.9)	0.017	0
Male	1490 (55.3)	2082 (56.1)		
Race				
Black	413 (15.3)	447 (12.0)	0.099	0
White	1777 (65.9)	2566 (69.2)		
Other^a^	104 (3.9)	157 (4.2)		
Unknown	401 (14.9)	540 (14.5)		
Ethnicity				
Hispanic	141 (5.3)	231 (6.2)	0.067	0.5
Non-Hispanic	2155 (80.4)	3003 (81.3)		
Unknown	385 (14.4)	461 (12.5)		
Admitted from home				
Yes	2377 (88.4)	2619 (70.7)	0.448	0.2
No	313 (11.6)	1083 (29.3)		
ASA class				
1-2	562 (20.9)	154 (4.2)	0.951	0.3
3	1335 (49.6)	1054 (28.5)		
4	702 (26.1)	1567 (42.4)		
5	94 (3.5)	920 (24.9)		
RAI, mean (SD)	23.41 (7.92)	26.98 (8.18)	0.444	25.4
Functional status				
Independent	2467 (92.5)	3080 (86.4)	0.211	2.7
Partially dependent	165 (6.2)	359 (10.1)		
Totally dependent	34 (1.3)	126 (3.5)		
BMI, mean (SD)^b^	29.61 (7.50)	28.55 (7.84)	0.137	8.3
Smoking	569 (21.1)	893 (24.1)	0.071	0
Ventilator dependence	63 (2.3)	692 (18.7)	0.552	0
Weight loss	73 (3.5)	136 (4.8)	0.066	23.5
Diabetes	366 (13.6)	452 (12.2)	0.042	0
Dyspnea	34 (1.6)	111 (3.9)	0.141	23.5
COPD	302 (11.2)	572 (15.4)	0.124	0
Sepsis or septic shock				
SIRS/sepsis	320 (11.9)	875 (23.6)	0.670	0
Septic shock	121 (4.5)	776 (20.9)		
Cancer	104 (3.9)	224 (6.0)	0.101	0
Ascites	24 (0.9)	155 (4.2)	0.210	0
Hypertension on medication	1758 (65.2)	2565 (69.1)	0.083	0
Heart failure	221 (8.2)	442 (11.9)	0.124	0
Transfusion^c^	146 (5.4)	692 (18.7)	0.415	0
Bleeding disorder	352 (13.1)	848 (22.9)	0.257	0
Dialysis	191 (7.1)	361 (9.7)	0.095	0
Acute kidney failure	52 (2.1)	236 (6.9)	0.235	8.3
Hematocrit, mean (SD), %	37.39 (6.89)	35.06 (7.87)	0.315	4.4
Platelets, mean (SD), 1000s/µL	244.12 (98.37)	221.59 (111.44)	0.214	4.9
White blood cells, mean (SD), 1000s/µL	8.92 (4.61)	12.16 (7.81)	0.504	5.4
Sodium, mean (SD), mEq/L	138.40 (3.78)	137.85 (5.00)	0.125	5.3
Blood urea nitrogen, mean (SD), mg/dL	23.03 (16.34)	30.68 (21.35)	0.402	7.3
Creatinine, mean (SD), mg/dL	1.52 (1.60)	1.85 (1.64)	0.201	4.7
Procedure urgency				
Elective	1722 (64.0)	973 (26.4)	0.911	0.5
Urgent	460 (17.1)	635 (17.2)		
Emergent	507 (18.9)	2077 (56.4)		
Operative stress score				
1-2	658 (25.5)	425 (12.1)	0.426	5.1
3	1335 (51.7)	1737 (49.6)		
4-5	588 (22.8)	1337 (38.2)		
ACS-NSQIP predicted mortality, mean (SD)^d^	0.05 (0.10)	0.22 (0.23)	0.946	0.0
2012-2017	1366 (50.7)	1844 (49.7)	0.020	0.0
2018-2023	1329 (49.3)	1866 (50.3)		

Abbreviations: ACS-NSQIP, American College of Surgeons National Surgical Quality Improvement Program; ASA, American Society of Anesthesiologists; BMI, body mass index; COPD, chronic obstructive pulmonary disease; RAI, Risk Analysis Index; SIRS, systemic inflammatory response syndrome; SMD, absolute standardized mean difference.

SI conversion factors: To convert creatinine to μmol/L, multiply by 88.4; hematocrit to proportion of 1.0, multiply by 0.01; platelet count to ×10^9^/μL, muliply by 1.0; sodium to mmol/L, multiply by 1.0; urea nitrogen to mmol/L, multiply by 0.357; white blood cell count to ×10^9^/L, multiply by 0.001.

^a^ Other race category includes American Indian or Alaska Native, Asian, Native Hawaiian or Pacific Islander, or some other race.

^b^ Calculated as weight in kilograms divided by height in meters squared.

^c^ Transfusion: at least 1 unit of packed red blood cells or whole blood in the 72 hours preoperation.

^d^ ACS-NSQIP predicted mortality: not included as predictor variable, but used as comparison for predictive models.

### Mortality

Of the 6405 patients included in the analysis, 3710 (57.9%) died within 30 days. All models demonstrated acceptable discrimination. AUROCs ranged from 0.78 (95% CI, 0.76-0.80) for the neural network to 0.80 (95% CI, 0.78-0.82) for AI, random forest, and ACS-NSQIP (Table 2). In terms of accuracy, random forest, LASSO regression, logistic regression, neural network, extreme gradient boosting, and support vector machine all showed comparable performance (accuracy range, 0.72; 95% CI, 0.70-0.74 for the neural network to 0.73; 95% CI, 0.71-0.75 for random forest, LASSO, logistic regression, extreme gradient boosting, and support vector machine) and outperformed naive Bayes and ACS-NSQIP predicted mortality (accuracy, 0.64; 95% CI, 0.62-0.66 and 0.50; 95% CI, 0.47-0.52, respectively). All models offered favorable combinations of sensitivity (range, 0.73; 95% CI, 0.71-0.76 for the neural network to 0.79; 95% CI, 0.76-0.81 for random forest and support vector machine) and specificity (range, 0.64; 95% CI, 0.61-0.68 for random forest to 0.70; 95% CI, 0.66-0.73 for the neural network), except for naive Bayes and ACS-NSQIP, which exhibited substantially lower sensitivity (0.45; 95% CI, 0.42-0.48 and 0.14; 95% CI, 0.12-0.16, respectively).

**Table 2.** Model Evaluation and Performance on Test Data

Model	AUROC (95% CI)	Accuracy (95% CI)	Sensitivity (95% CI)	Specificity (95% CI)	PPV (95% CI)	NPV (95% CI)	Brier Score
30-d Mortality							
Extreme gradient boosting	0.80 (0.78-0.82)	0.73 (0.71-0.75)	0.77 (0.74-0.79)	0.68 (0.65-0.71)	0.77 (0.74-0.79)	0.68 (0.64-0.71)	0.18
Random forest	0.80 (0.78-0.82)	0.73 (0.71-0.75)	0.79 (0.76-0.81)	0.64 (0.61-0.68)	0.75 (0.73-0.78)	0.69 (0.65-0.72)	0.18
Logistic regression	0.79 (0.77-0.81)	0.73 (0.71-0.75)	0.77 (0.74-0.79)	0.68 (0.65-0.71)	0.77 (0.74-0.79)	0.68 (0.75-0.71)	0.18
LASSO	0.79 (0.77-0.81)	0.73 (0.71-0.75)	0.77 (0.74-0.79)	0.67 (0.64-0.71)	0.76 (0.74-0.79)	0.68 (0.64-0.71)	0.18
Support vector machine	0.79 (0.77-0.81)	0.73 (0.71-0.75)	0.79 (0.76-0.81)	0.65 (0.62-0.69)	0.76 (0.73-0.78)	0.69 (0.66-0.72)	0.18
Neural network	0.78 (0.76-0.80)	0.72 (0.70-0.74)	0.73 (0.71-0.76)	0.70 (0.66-0.73)	0.77 (0.74-0.79)	0.66 (0.62-0.69)	0.19
Naive Bayes	0.79 (0.77-0.81)	0.64 (0.62-0.66)	0.45 (0.42-0.48)	0.90 (0.88-0.92)	0.86 (0.83-0.89)	0.54 (0.52-0.57)	0.31
ACS-NSQIP	0.80 (0.78-0.82)	0.50 (0.47-0.52)	0.14 (0.12-0.16)	0.99 (0.98-1.00)	0.95 (0.90-0.98)	0.45 (0.43-0.48)	0.40
Nonhome discharge							
Extreme gradient boosting	0.78 (0.74-0.82)	0.76 (0.74-0.80)	0.48 (0.42-0.55)	0.91 (0.88-0.93)	0.72 (0.64-0.78)	0.78 (0.75-0.81)	0.17
Random forest	0.79 (0.76-0.83)	0.76 (0.73-0.79)	0.40 (0.34-0.47)	0.94 (0.91-0.96)	0.77 (0.69-0.84)	0.76 (0.73-0.79)	0.17
Logistic regression	0.76 (0.73-0.80)	0.76 (0.73-0.79)	0.48 (0.42-0.54)	0.90 (0.87-0.92)	0.70 (0.63-0.77)	0.78 (0.74-0.81)	0.17
LASSO	0.77 (0.73-0.80)	0.76 (0.73-0.79)	0.48 (0.42-0.55)	0.90 (0.87-0.93)	0.71 (0.64-0.78)	0.78 (0.74-0.81)	0.17
Support vector machine	0.78 (0.75-0.82)	0.75 (0.72-0.78)	0.54 (0.48-0.60)	0.86 (0.83-0.89)	0.65 (0.58-0.72)	0.79 (0.75-0.82)	0.17
Neural network	0.76 (0.73-0.80)	0.76 (0.73-0.79)	0.50 (0.44-0.57)	0.88 (0.85-0.91)	0.68 (0.61-0.75)	0.78 (0.75-0.82)	0.17
Naive Bayes	0.74 (0.71-0.78)	0.72 (0.69-0.75)	0.48 (0.41-0.54)	0.84 (0.80-0.87)	0.59 (0.52-0.66)	0.76 (0.73-0.80)	0.25
ACS-NSQIP	0.78 (0.74-0.81)	0.68 (0.64-0.71)	0.03 (0.01-0.06)	1.00 (0.99-1.00)	0.89 (0.52-1.00)	0.68 (0.64-0.71)	0.28

Abbreviations: ACS-NSQIP, American College of Surgeons National Safety and Quality Improvement Program predicted mortality; AUROC, area under the receiver operating characteristic curve; LASSO, least absolute shrinkage and selection operator; NPV, negative predictive value; PPV, positive predictive value.

**Figure 1.**
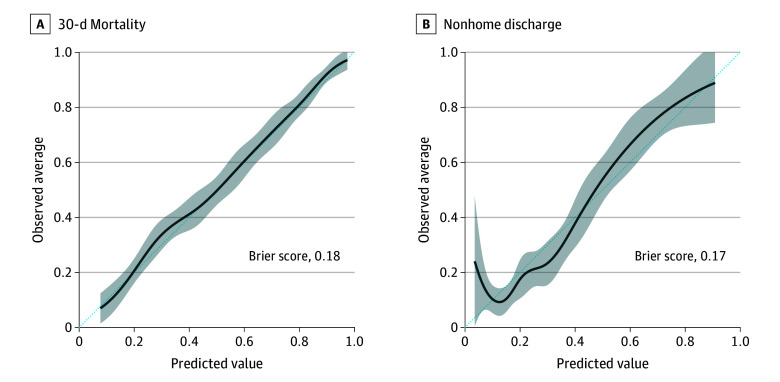
Calibration Plots for the Extreme Gradient Boosting Model Predicting 30-Day Mortality and Nonhome Discharge After Perioperative Cardiopulmonary Resuscitation The diagonal line represents perfect calibration where predicted probabilities equal observed outcomes. Shaded areas indicate 95% CIs. Points above the diagonal indicate that the model underestimates the true probability, whereas points below the diagonal indicate overestimation. The Brier score quantifies the discrepancy between predicted probabilities and actual outcomes, with lower values indicating better calibration.

Brier scores likewise indicated acceptable calibration (0.18-0.19) across all models except for naive Bayes (0.31) and ACS-NSQIP (0.40) (Figure 1A). Overall, extreme gradient boosting offered the most favorable combination of performance characteristics. Subsequent discussion will therefore focus on extreme gradient boosting.

**Figure 2.**
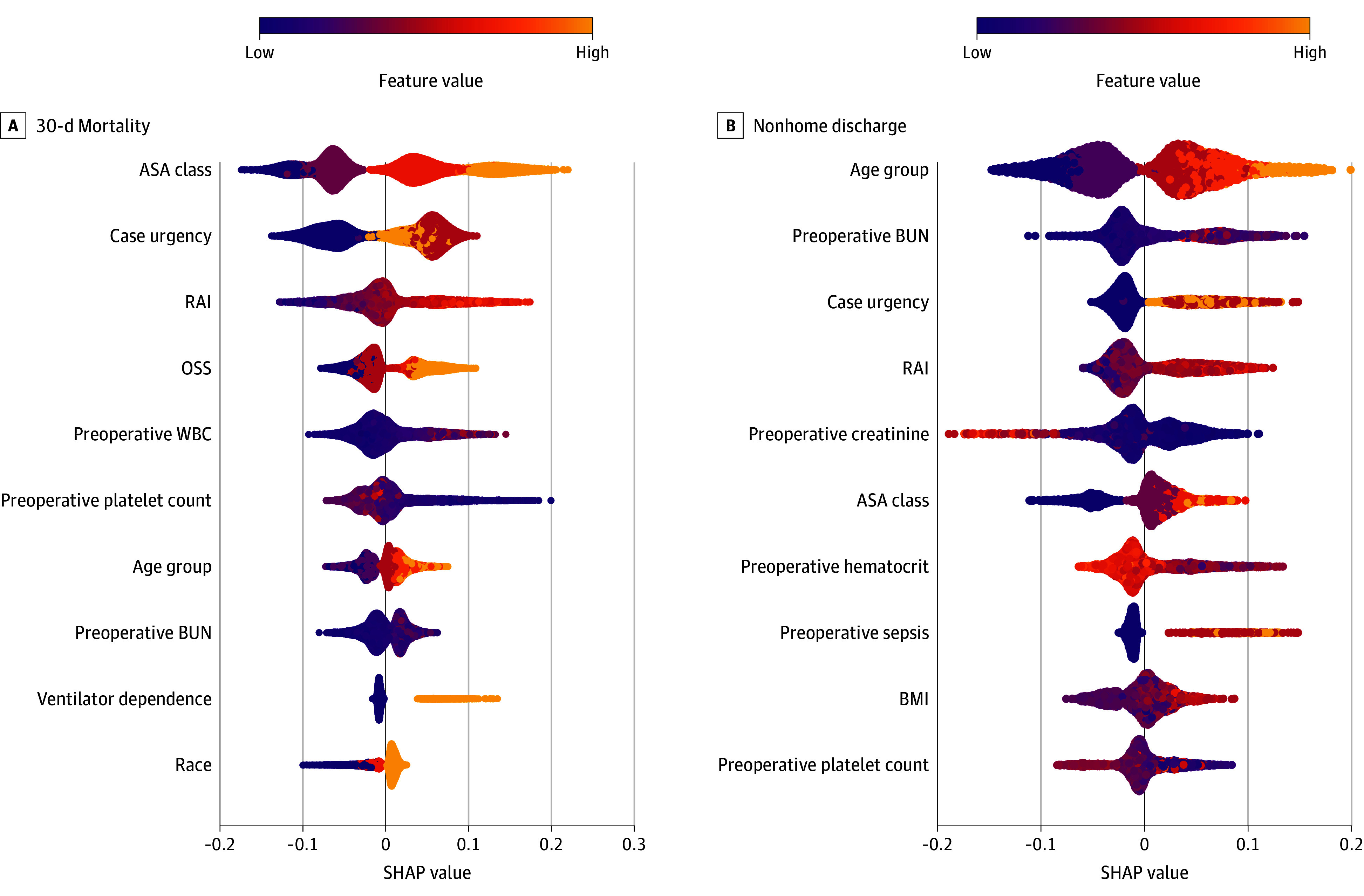
Shapley Additive Explanation (SHAP) Plots Illustrating the Contribution of Clinical Variables to Predictions of 30-Day Mortality and Nonhome Discharge Following Perioperative Cardiopulmonary Resuscitation Variables are listed from top to bottom according to their predictive importance. Each point represents an individual observation with SHAP values indicating the contribution to the prediction: positive values (to the right) increase the probability of the outcome, while negative values (to the left) decrease it. Colors indicate the variable’s value, from low (purple) to high (yellow). For instance, higher ASA class (shown in yellow) strongly increased predicted mortality, whereas White race slightly increased predicted mortality. ASA indicates American Society of Anesthesiologists; BMI, body mass index; BUN, blood urea nitrogen; OSS, operative stress score; RAI, Risk Analysis Index; WBC, white blood cell count.

**Figure 3.**
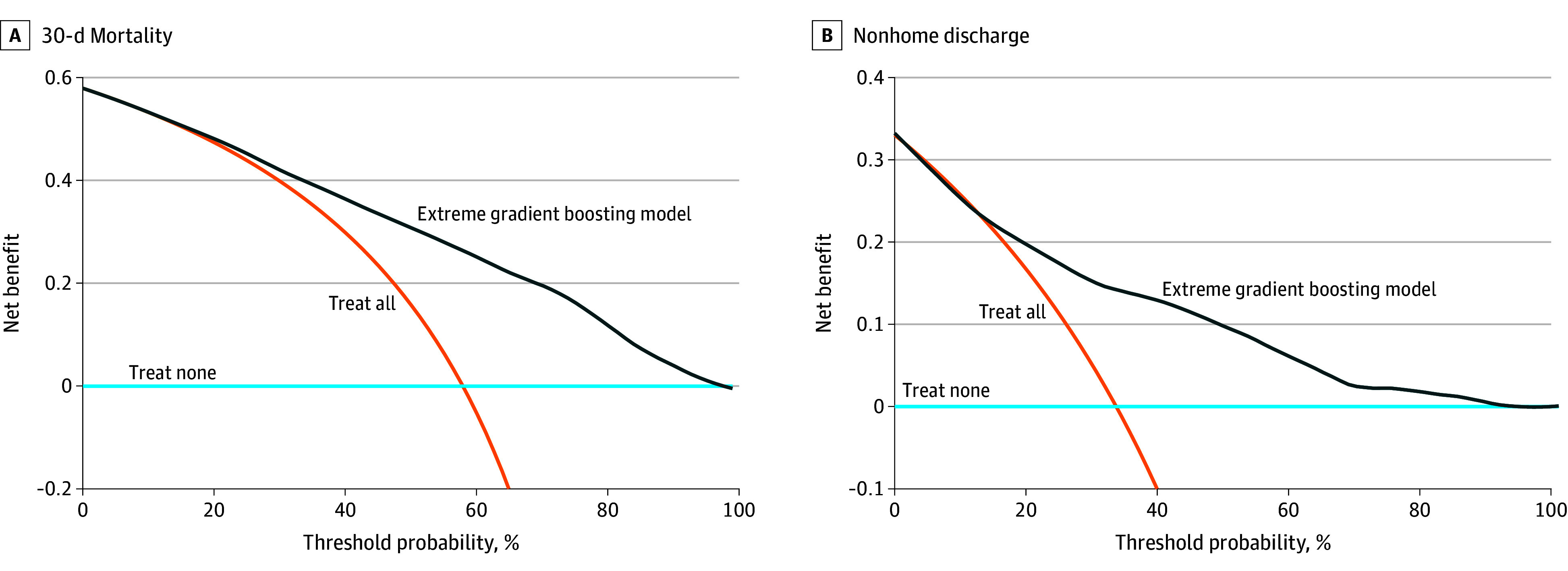
Decision Curve Analyses for the Extreme Gradient Boosting Models Predicting 30-Day Mortality and Nonhome Discharge Following Perioperative Cardiopulmonary Resuscitation Decision curve analysis evaluates the clinical usefulness of a predictive model by characterizing the value of the information it provides across a range of preferences regarding the relative burdens of undertreatment vs overtreatment. Such preferences are represented by the threshold probability (plotted on the x-axis), defined here as the predicted probability of a negative outcome beyond which a patient or clinician may reconsider the decision to proceed with cardiopulmonary resuscitation. The value of the information provided by the model at each threshold is quantified by net benefit (plotted on the y-axis), calculated as the proportion of true positives minus the proportion of false positives, weighted by the odds of the selected threshold probability.

SHAP values for extreme gradient boosting identified ASA physical status and case urgency as the most influential predictors of 30-day mortality (Figure 2A). Higher ASA classes (eg, 4-5) and emergent case status were associated with positive SHAP values, indicating increased predicted mortality risk. Other highly ranked predictors included the RAI (frailty), OSS, age, and ventilator dependence. Laboratory values—WBC, BUN, and platelet count—exhibited mixed SHAP effects, with both positive and negative contributions across their ranges, suggesting their influence may vary in interaction with other clinical features. White race was associated with slightly higher predicted mortality, though the magnitude of this effect was small compared with other variables. DCA demonstrated that the model provided greater net benefit than default strategies (treat all or treat none) across a wide range of threshold probabilities (approximately 10% to 90%), with the greatest differences observed between 50% and 80% (Figure 3A).

### Nonhome Discharge

Among the 2478 patients who were admitted from home and survived to discharge, 822 (33.2%) were discharged to a destination other than home. All models demonstrated acceptable discrimination for predicting nonhome discharge, with AUROC values ranging from 0.74 (95% CI, 0.71-0.78) for naive Bayes to 0.79 (95% CI, 0.76-0.83) for random forest and accuracy ranging from 0.68 (95% CI, 0.64-0.71) for ACS-NSQIP to 0.76 (95% CI, 0.74-0.80) for extreme gradient boosting, random forest, logistic regression, LASSO, and the neural network. Performance was associated with greater specificity (range, 0.84; 95% CI, 0.80-0.87 for naive Bayes to 1.00; 95% CI, 0.99-1.00 for ACS-NSQIP) but lower sensitivity (range, 0.03; 95% CI, 0.01-0.06 for ACS-NSQIP to 0.54; 95% CI, 0.48-0.60), relative to performance in the mortality prediction models. Calibration was comparable to the mortality models, with Brier scores of 0.17 for all models except naive Bayes (0.25) and ACS-NSQIP (0.28) (Figure 1B). As with mortality prediction, extreme gradient boosting offered the most favorable combination of performance characteristics for nonhome discharge.

SHAP values indicated that ASA status, case urgency, and frailty were important contributors to predictions of nonhome discharge. Chronological age played a greater role in this context than in mortality prediction, particularly among patients with high predicted probabilities of nonhome discharge (Figure 2B). DCA for nonhome discharge showed that the model provided greater net benefit than default strategies (treat all or treat none) across threshold probabilities of approximately 12% to 90%, with the largest gains observed between 30% and 60% (Figure 3B).

### Sensitivity Analysis

Results were not sensitive to excluding 2021 to 2023 data due to 3 missing variables. Detailed results of the sensitivity analysis are available in eTable 2 in Supplement 1.

## Discussion

In this analysis of 6405 patients from the ACS-NSQIP who underwent CPR for perioperative cardiac arrest between 2012 and 2023, we developed and validated models predicting 30-day mortality and nonhome discharge using routinely available preoperative data. Extreme gradient boosting demonstrated the best overall performance. To our knowledge, extreme gradient boosting is the first tool designed specifically to predict individual outcomes following perioperative CPR. While scoring systems for in-hospital cardiac arrest have existed for more than a decade,^40,41^ literature on perioperative CPR outcomes has historically focused on population-level associations and general risk estimates.^3^ Extreme gradient boosting instead clarifies the prognostic significance of clinical features and generates individualized risk assessments, providing a necessary basis for primary prevention strategies and SDM regarding the appropriateness of perioperative CPR.

Among the candidate variables, ASA physical status and frailty emerged as important predictors of mortality following perioperative CPR. This finding is consistent with studies demonstrating an association between these variables and unfavorable outcomes.^2,4,18^ However, earlier work suggested that incorporating frailty into baseline models did not significantly improve performance.^19^ Notably, that study employed logistic regression, which is less capable of capturing complex, nonlinear relationships and interactions between predictors. The importance of frailty in shaping extreme gradient boosting predictions suggests that frailty’s prognostic value may have been underestimated previously due to methodological limitations.

Compared with ASA physical status and frailty severity, chronological age was less important in influencing model predictions of mortality. Context-specific evidence regarding the predictive importance of chronological age is critical to limit bias in decision-making regarding appropriateness of life-sustaining therapies (LSTs),^42,43,44^ and our results indicate that age alone should not be used to gauge likelihood of survival following perioperative CPR. Physicians should instead consider age in relation to measures of comorbidity severity (ie, ASA status), physiologic reserve (ie, frailty), and clinical context (ie, OSS and case urgency).

In contrast, age was one of the most important factors predicting nonhome discharge among survivors of perioperative cardiac arrest, likely reflecting selective survival given high mortality among patients with severe frailty.^2^ Although nonhome discharge is an imperfect proxy for functional recovery, our findings support concerns that older survivors may face extended recovery and potential loss of functional independence.^45^ Frailty also contributed to predictions of nonhome discharge, highlighting the need for studies examining longer-term functional trajectory after perioperative CPR.^15,46,47^

Extreme gradient boosting predictions may inform strategies aimed at preventing cardiac arrest in the highest-risk patients. For some, this could include consideration of less invasive surgical approaches or nonoperative management.^13,48^ When surgery is undertaken, measures such as preoperative optimization, robust anesthesia staffing, and postoperative intensive care unit admission may reduce risk of cardiac arrest.^1^ Because these interventions are resource-intensive, evidence-based risk stratification is essential to guide their appropriate allocation.

As efforts to align surgical care with what matters most to patients expand, tools like extreme gradient boosting can play an important role.^49^ Extreme gradient boosting may help identify patients for whom code status discussions are particularly salient by providing individualized risk estimates.^20,50^ For example, perceived futility of CPR may motivate patients and practitioners to maintain do not resuscitate (DNR) orders perioperatively.^16^ If based on inaccurate assumptions about likely outcomes, such judgments can lead to withholding of treatments that may actually align with patients’ priorities. Conversely, routinely suspending DNR orders because of expected reversibility of perioperative cardiac arrest may expose patients to treatment burdens they would deem unacceptable.^12,13,15,51^

By grounding discussions in individualized risk predictions rather than general assumptions about CPR outcomes, extreme gradient boosting has the potential to enhance the accuracy and integrity of SDM focused on patients’ unique concerns and circumstances.^20,50,51,52,53^ However, implementation will require development of a structured communication tool in collaboration with diverse stakeholders and in accordance with established standards for patient decision aids.^54,55^ This process is necessary to integrate predictive information into clinical care in a way that meets the needs of high-risk patients, care partners, and clinicians.

While the performance of extreme gradient boosting is comparable with that of risk calculators used in other contexts,^40,41^ its limitations reflect challenges inherent to predicting outcomes following perioperative CPR. Integration of preoperative data from electronic health records^24,26^ and additional biomarkers^56,57,58^ are logical areas for future study, but resulting improvements in predictive performance are likely to be marginal. Residual uncertainty likely stems not from limitations of preoperative data, but from the heterogeneity of perioperative cardiac arrest and resuscitation.^59^

For example, studies have demonstrated that clinical context, arrest cause, and resuscitation characteristics play a significant role in shaping outcomes.^5,60,61,62,63^ In one recent investigation incorporating baseline patient data and intraoperative variables, the most important predictor of mortality was CPR duration exceeding 60 minutes. Other key features included massive hemorrhage or transfusion, arrest cause, and cumulative epinephrine dose.^27^ This evidence highlights the need to supplement preoperative risk stratification with dynamic prognostic assessments.

Preoperative risk assessments and code status discussions are important, but SDM regarding perioperative LSTs is best understood as a longitudinal process guided by preoperative, intraoperative, and postoperative data in conversation with patients’ goals, values, and evolving clinical status.^64,65,66^ When faced with prognostic uncertainty, preoperative planning for time-limited trials (TLTs) in the event of perioperative cardiac arrest is a pragmatic approach to decision-making regarding perioperative CPR.^20,64^ Defined as a plan to use LST for a defined duration, assess response to therapy, and then decide whether to continue LST, shift to comfort care, or prolong the trial,^67^ the TLT is increasingly recognized as a means of addressing prognostic uncertainty and aligning care with patients’ priorities in critical illness.^68,69,70,71,72^

### Strengths and Limitations

This investigation has several notable strengths. To our knowledge, this is the largest cohort ever studied to investigate outcomes following perioperative cardiac arrest, and the integrity of the NSQIP dataset is well-established. The inclusion of readily available demographic, clinical, laboratory, and surgical variables balances granularity with generalizability and feasibility. Methodologically, this work is an important advance beyond existing literature in clarifying the prognostic importance of clinical features and generating patient-specific predictions of mortality and disposition.

This study also has several limitations. External validation of our models was not feasible due to unavailability of appropriate datasets. While variables not included in the dataset might enhance performance, their absence reflects a tradeoff favoring generalizability. Finally, the 30-day follow-up period precludes analysis of patient-centered outcomes such as quality of life and days alive and out of hospital.^45,72,73^ Future studies should examine longer-term outcomes that patients value and integrate data from the intraoperative and postoperative period to inform future risk prediction tools.

## Conclusions

In this prospective prognostic study of outcomes following perioperative CPR, we developed and validated a tool to predict 30-day mortality and nonhome discharge. Individualized predictions may guide allocation of preventive strategies and support SDM regarding perioperative CPR. Realizing this potential will require integration into perioperative workflows and the development of decision aids that address the decision-making needs of patients, care partners, and clinicians.

## Notes

Supplement 1. eFigure. Study Flow Diagram eTable 1. Patient Characteristics and Candidate Predictors for Nonhome Discharge Following Perioperative Cardiopulmonary Resuscitation eTable 2. Model Evaluation and Performance on Test Data: Sensitivity Analysis Excluding 2021-2023 Data

Supplement 2. Data Sharing Statement
