# Supplementary material for: Prediction of Cardiopulmonary Resuscitation Outcomes for Arrest in Surgical Settings
Source: JAMA Netw Open. 2025 Oct 28;8(10):e2539767. doi: 10.1001/jamanetworkopen.2025.39767 (PMC12569717; doi:10.1001/jamanetworkopen.2025.39767)
Supplement: Supplement 1. — eFigure. Study Flow Diagram eTable 1. Patient Characteristics and Candidate Predictors for Nonhome Discharge Following Perioperative Cardiopulmonary Resuscitation eTable 2. Model Evaluation and Performance on Test Data: Sensitivity Analysis Excluding 2021-2023 Data [file jamanetwopen-e2539767-s001.pdf]

Supplemental Online Content

Chen L, Justice S, Allen MB. Cardiopulmonary resuscitation outcomes prediction for arrest in surgical setting. *JAMA Netw Open*. 2025;8(10):e2539767. doi:10.1001/jamanetworkopen.2025.39767

eFigure. Study Flow Diagram
eTable 1. Patient Characteristics and Candidate Predictors for Nonhome Discharge Following Perioperative Cardiopulmonary Resuscitation
eTable 2. Model Evaluation and Performance on Test Data: Sensitivity Analysis Excluding 2021-2023 Data

This supplemental material has been provided by the authors to give readers additional information about their work.

© 2025 Chen L et al. *JAMA Network Open.*

eFigure. Study Flow Diagram

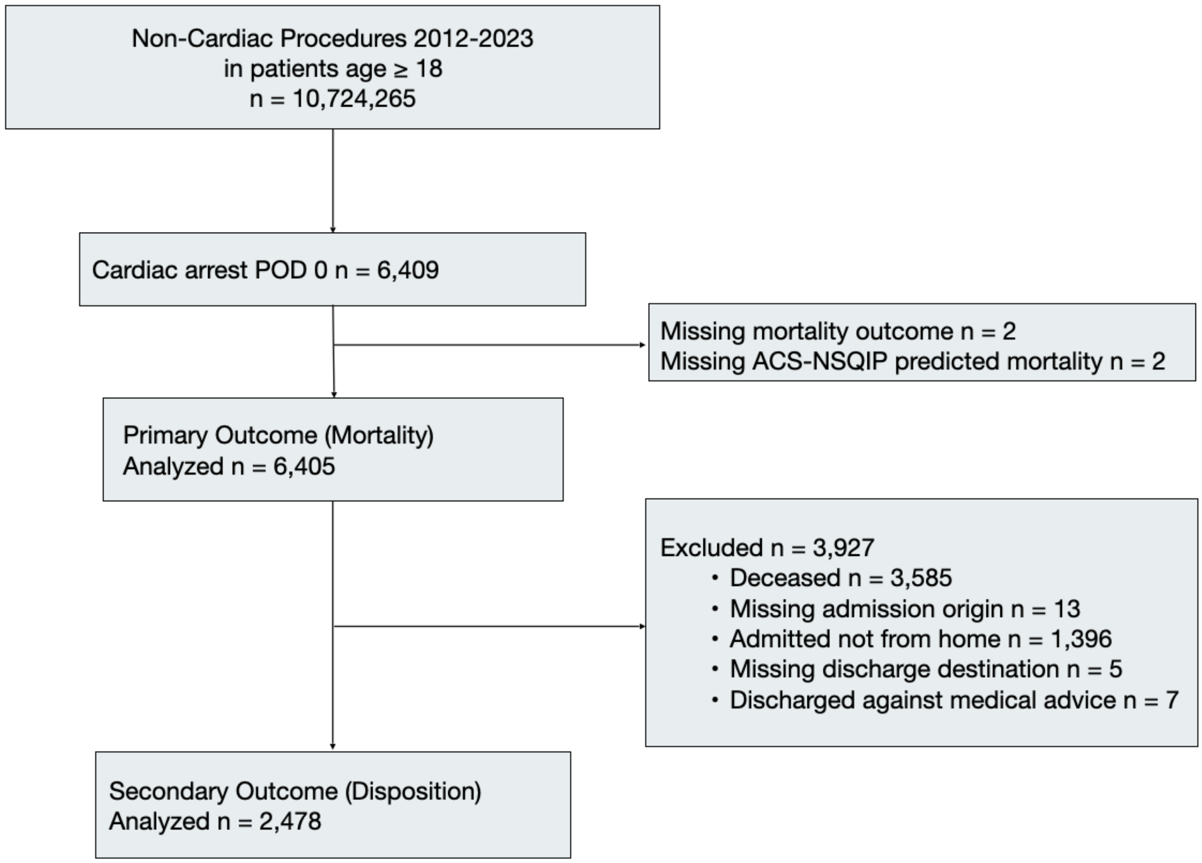

Abbreviations: POD = postoperative day; ACS-NSQIP = American College of Surgeons National Safety and Quality Improvement Program

© 2025 Chen L et al. *JAMA Network Open.*

eTable 1. Patient Characteristics and Candidate Predictors for Nonhome Discharge Following Perioperative Cardiopulmonary Resuscitation

Variable		Home, No. (%)	Non-Home, No. (%)	SMD	Missing (%)
n		1656	822		
Age	18-49	301 (18.2)	56 (6.8)	0.606	0.000
	50-64	598 (36.1)	193 (23.5)		
	65-74	434 (26.2)	249 (30.3)		
	75-84	261 (15.8)	200 (24.3)		
	85	62 (3.7)	124 (15.1)		
Sex	Female	728 (44.0)	386 (47.0)	0.060	0.0
	Male	928 (56.0)	436 (53.0)		
Race	Black	221 (13.3)	155 (18.9)	0.267	0.0
	White	1084 (65.5)	557 (67.8)		
	Other[a]	60 (3.6)	33 (4.0)		
	Unknown	291 (17.6)	77 (9.4)		
Ethnicity	Hispanic	90 (5.5)	47 (5.7)	0.231	0.4
	Non-Hispanic	1280 (77.7)	698 (85.1)		
	Unknown	278 (16.9)	75 (9.1)		
ASA Class	1-2	475 (28.7)	83 (10.1)	0.594	0.1
	3	854 (51.6)	405 (49.3)		
	4	293 (17.7)	296 (36.1)		
	5	33 (2.0)	37 (4.5)		
RAI, mean (SD)		21.49 (7.38)	26.17 (7.65)	0.623	24.7
Functional Status	Independent	1579 (96.3)	717 (88.2)	0.307	1.0
	Partially Dependent	54 (3.3)	81 (10.0)		
	Totally Dependent	7 (0.4)	15 (1.8)		

© 2025 Chen L et al. *JAMA Network Open.*

**Patient characteristics and candidate predictors for non-home discharge following perioperative cardiopulmonary resuscitation (Continued)**					
Variable		Home, No. (%)	Non-Home, No. (%)	SMD	Missing (%)
BMI, mean (SD)		29.91 (7.27)	29.13 (7.64)	0.104	2.5
Smoking		338 (20.4)	169 (20.6)	0.004	0.0
Ventilator dependence		14 (0.8)	24 (2.9)	0.153	0.0
Weight loss		34 (2.8)	34 (5.2)	0.122	24.1
Diabetes		185 (11.2)	143 (17.4)	0.179	0.0
Dyspnea		15 (1.2)	18 (2.7)	0.108	24.1
COPD		139 (8.4)	120 (14.6)	0.195	0.0
Sepsis or septic shock	SIRS/Sepsis	102 (6.2)	156 (19.0)	0.502	0.0
	Septic Shock	30 (1.8)	60 (7.3)		
Cancer		62 (3.7)	36 (4.4)	0.032	0.0
Ascites		12 (0.7)	11 (1.3)	0.061	0.0
Hypertension on medication		1010 (61.0)	596 (72.5)	0.246	0.0
Heart failure		104 (6.3)	96 (11.7)	0.190	0.0
Transfusion[b]		47 (2.8)	53 (6.4)	0.172	0.0
Bleeding disorder		160 (9.7)	144 (17.5)	0.231	0.0
Dialysis		95 (5.7)	79 (9.6)	0.146	0.0
Acute renal failure		19 (1.3)	26 (3.4)	0.142	8.2
Hematocrit, mean (SD), %		38.78 (6.19)	36.03 (7.17)	0.411	6.6
Platelets, mean (SD), 1,000s/μL		245.85 (88.87)	242.74 (106.07)	0.032	7.2
White Blood Cells, mean (SD), 1,000s/μL		8.08 (3.42)	9.74 (5.79)	0.347	7.3

© 2025 Chen L et al. *JAMA Network Open.*

Patient characteristics and candidate predictors for non-home discharge following perioperative cardiopulmonary resuscitation (Continued)					
Variable		Home, No. (%)	Non-Home, No. (%)	SMD	Missing (%)
Sodium, mean (SD), mEq/L		138.77 (3.35)	138.05 (4.12)	0.191	8.2
Blood Urea Nitrogen , mean (SD), mg/dL		20.39 (14.00)	26.35 (17.60)	0.375	10.8
Creatinine, mean (SD), mg/dL		1.42 (1.60)	1.67 (1.65)	0.153	7.2
Procedure Urgency	Elective	1314 (79.5)	409 (49.9)	0.653	0.2
	Urgent	165 (10.0)	204 (24.9)		
	Emergent	173 (10.5)	207 (25.2)		
Operative Stress Score	1-2	482 (30.7)	159 (19.8)	0.254	4.2
	3	751 (47.8)	453 (56.4)		
	4-5	338 (21.5)	191 (23.8)		
ACS-NSQIP Predicted Mortality[c], mean (SD)		0.02 (0.06)	0.08 (0.12)	0.563	0.0
Year	2012-2017	766 (46.3)	454 (55.2)	0.180	0.0
	2018-2023	890 (53.7)	368 (44.8)		

Notes: Abbreviations: SMD = absolute standardized mean difference, SD = standard deviation, ASA = American Society of Anesthesiologists, RAI = risk analysis index, BMI = Body Mass Index, COPD = chronic obstructive pulmonary disease, ACS-NSQIP = American College of Surgeons National Surgical Quality Improvement Program.
[a]Other race category includes American Indian or Alaska Native, Asian, Native Hawaiian or Pacific Islander, or some other race.
[b]Transfusion: at least one unit of packed red blood cells or whole blood in the 72 hours pre-operation.
[c]ACS-NSQIP Predicted Mortality: Not included as predictor variable, but used as comparison for predictive models.

© 2025 Chen L et al. *JAMA Network Open.*

eTable 2. Model Evaluation and Performance on Test Data: Sensitivity Analysis Excluding 2021-2023 Data

Model	AUROC (95% CI)	Accuracy (95% CI)	Sensitivity (95% CI)	Specificity (95% CI)	PPV (95% CI)	NPV (95% CI)	Brier Score
30-Day Mortality							
XGBoost	0.79 (0.77-0.82)	0.72 (0.70-0.74)	0.77 (0.74-0.79)	0.66 (0.62-0.69)	0.75 (0.72-0.78)	0.68 (0.64-0.71)	0.18
Random Forest	0.80 (0.77-0.82)	0.72 (0.69-0.74)	0.77 (0.74-0.80)	0.64 (0.60-0.68)	0.74 (0.71-0.77)	0.67 (0.63-0.71)	0.18
Logistic Regression	0.79 (0.76-0.81)	0.72 (0.70-0.75)	0.77 (0.74-0.79)	0.67 (0.63-0.70)	0.76 (0.73-0.79)	0.68 (0.64-0.71)	0.19
LASSO	0.79 (0.76-0.81)	0.72 (0.69-0.74)	0.77 (0.74-0.79)	0.65 (0.61-0.68)	0.75 (0.72-0.77)	0.67 (0.63-0.71)	0.19
Support Vector Machine	0.78 (0.75-0.80)	0.72 (0.70-0.74)	0.78 (0.75-0.81)	0.64 (0.60-0.68)	0.75 (0.72-0.77)	0.68 (0.64-0.72)	0.19
Neural Network	0.77 (0.75-0.79)	0.72 (0.70-0.75)	0.74 (0.71-0.77)	0.70 (0.66-0.73)	0.77 (0.74-0.80)	0.67 (0.63-0.70)	0.19
Naïve Bayes	0.79 (0.77-0.81)	0.57 (0.54-0.59)	0.27 (0.24-0.31)	0.96 (0.95-0.98)	0.91 (0.87-0.94)	0.50 (0.47-0.52)	0.38
ACS-NSQIP	0.79 (0.76-0.81)	0.49 (0.46-0.52)	0.12 (0.10-0.15)	0.99 (0.98-1.00)	0.94 (0.87-0.97)	0.45 (0.43-0.48)	0.40

© 2025 Chen L et al. *JAMA Network Open.*

Model Evaluation and Performance on Test Data - Sensitivity Analysis Excluding 2021-2023 Data (Continued)

Model	AUROC (95% CI)	Accuracy (95% CI)	Sensitivity (95% CI)	Specificity (95% CI)	PPV (95% CI)	NPV (95% CI)	Brier Score
Non-Home Discharge							
XGBoost	0.75 (0.71-0.79)	0.73 (0.69-0.77)	0.41 (0.34-0.48)	0.90 (0.87-0.93)	0.70 (0.61-0.78)	0.74 (0.69-0.78)	0.23
Random Forest	0.74 (0.70-0.79)	0.74 (0.70-0.78)	0.45 (0.38-0.52)	0.90 (0.87-0.93)	0.72 (0.63-0.79)	0.75 (0.71-0.79)	0.19
Logistic Regression	0.75 (0.70-0.79)	0.74 (0.70-0.77)	0.45 (0.38-0.52)	0.90 (0.86-0.93)	0.70 (0.61-0.78)	0.75 (0.70-0.79)	0.19
LASSO	0.75 (0.70-0.79)	0.73 (0.69-0.77)	0.41 (0.34-0.48)	0.90 (0.87-0.93)	0.70 (0.61-0.78)	0.74 (0.69-0.78)	0.19
Support Vector Machine	0.74 (0.70-0.78)	0.74 (0.70-0.77)	0.46 (0.39-0.53)	0.88 (0.85-0.92)	0.69 (0.60-0.76)	0.75 (0.71-0.79)	0.19
Neural Network	0.74 (0.70-0.79)	0.72 (0.68-0.76)	0.50 (0.43-0.57)	0.84 (0.80-0.88)	0.64 (0.56-0.71)	0.76 (0.71-0.80)	0.19
Naïve Bayes	0.74 (0.69-0.78)	0.69 (0.65-0.73)	0.17 (0.12-0.23)	0.98 (0.96-0.99)	0.81 (0.66-0.91)	0.68 (0.64-0.72)	0.29
ACS-NSQIP	0.73 (0.69-0.77)	0.66 (0.62-0.70)	0.03 (0.01-0.06)	1.00 (0.99-1.00)	1.00 (0.48-1.00)	0.65 (0.61-0.69)	0.32

AUROC = area under the receiver operating characteristic curve
PPV = positive predictive value
NPV = negative predictive value
XGBoost = extreme gradient boosting
LASSO = least absolute shrinkage and selection operator
ACS-NSQIP = American College of Surgeons National Safety and Quality Improvement Program predicted mortality

© 2025 Chen L et al. *JAMA Network Open.*
