# Supplementary material for: Prediction of Cardiopulmonary Resuscitation Outcomes for Arrest in Surgical Settings
Source: JAMA Netw Open. 2025 Oct 28;8(10):e2539767. doi: 10.1001/jamanetworkopen.2025.39767 (PMC12569717; doi:10.1001/jamanetworkopen.2025.39767)
Supplement: Supplement 2. — Data Sharing Statement [file jamanetwopen-e2539767-s002.pdf]

Data Sharing Statement

Chen. Cardiopulmonary Resuscitation Outcomes Prediction for Arrest in Surgical Settings.
JAMA Netw Open. Published October 28, 2025. doi:10.1001/jamanetworkopen.2025.39767

Data
Data available: No

Additional Information
Explanation for why data not available: The data are available from the ACS-NSQIP.
